# Supplementary material for: In vivo imaging with a fast large-area multiphoton exoscope (FLAME) captures the melanin distribution heterogeneity in human skin
Source: Sci Rep. 2022 May 16;12:8106. doi: 10.1038/s41598-022-12317-y (PMC9110384; doi:10.1038/s41598-022-12317-y)
Supplement: Supplementary file 1 — Supplementary Information. [file 41598_2022_12317_MOESM1_ESM.pdf]

SUPPLEMENTARY DATA

In vivo imaging with a fast large-area multiphoton exoscope (FLAME) captures the melanin distribution heterogeneity in human skin

Juvinch R. Vicente[1], *Amanda Durkin*[1], *Kristina Shrestha*[1], *Mihaela Balu*[1]*

[1]Beckman Laser Institute and Medical Clinic, University of California, Irvine, 1002 Health Sciences Rd., Irvine, CA 92612, USA

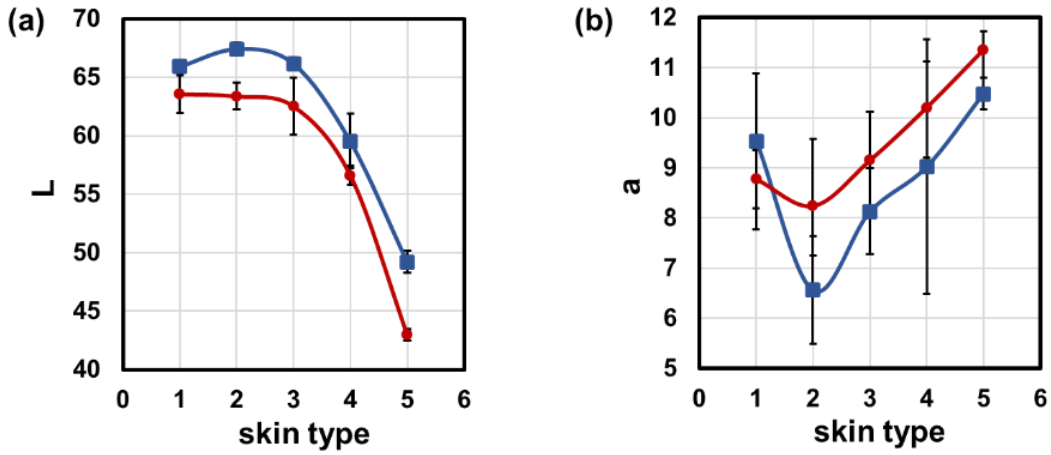

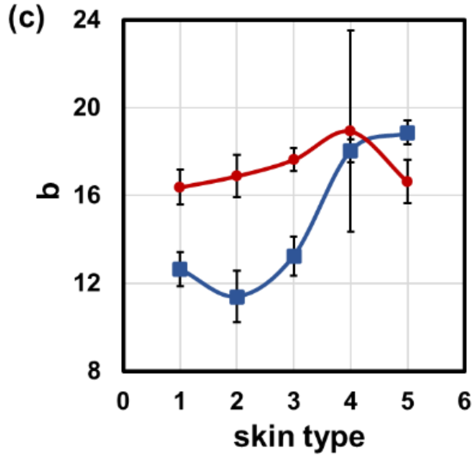

(d)

Correlation Coefficients

	*L**	*a**	*b**
Volar, MVF	-0.89	0.51	0.94
Dorsal, MVF	-0.78	0.78	0.68

Figure S1. Summary of colorimetry measurements. (a, b, c) The lightness (L*), *a**, and *b** parameters from colorimetry measurements for each skin phototype. The data points and error bar in each plot correspond to the average and standard deviation of each measurement, respectively. (N=6). The blue and red curves correspond to volar and dorsal forearms, respectively. (d) The correlation matrix of the colorimetric parameters and the melanin volume fractions (MVF).

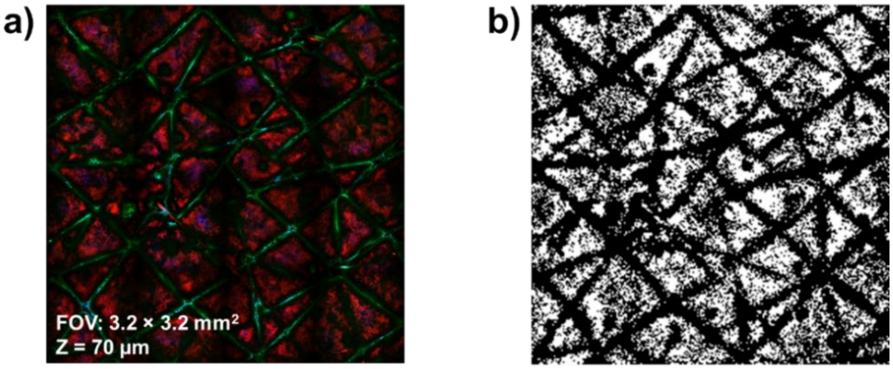

Figure S2. (a) An example of a raw 3-channel intensity images acquired in real-time with FLAME. (b) The equivalent melanin binary image obtained from the difference in the red and green channels, where thresholding was used to set pixels with difference in intensity > 0 to 1, and difference of ≤ 0 to 0.

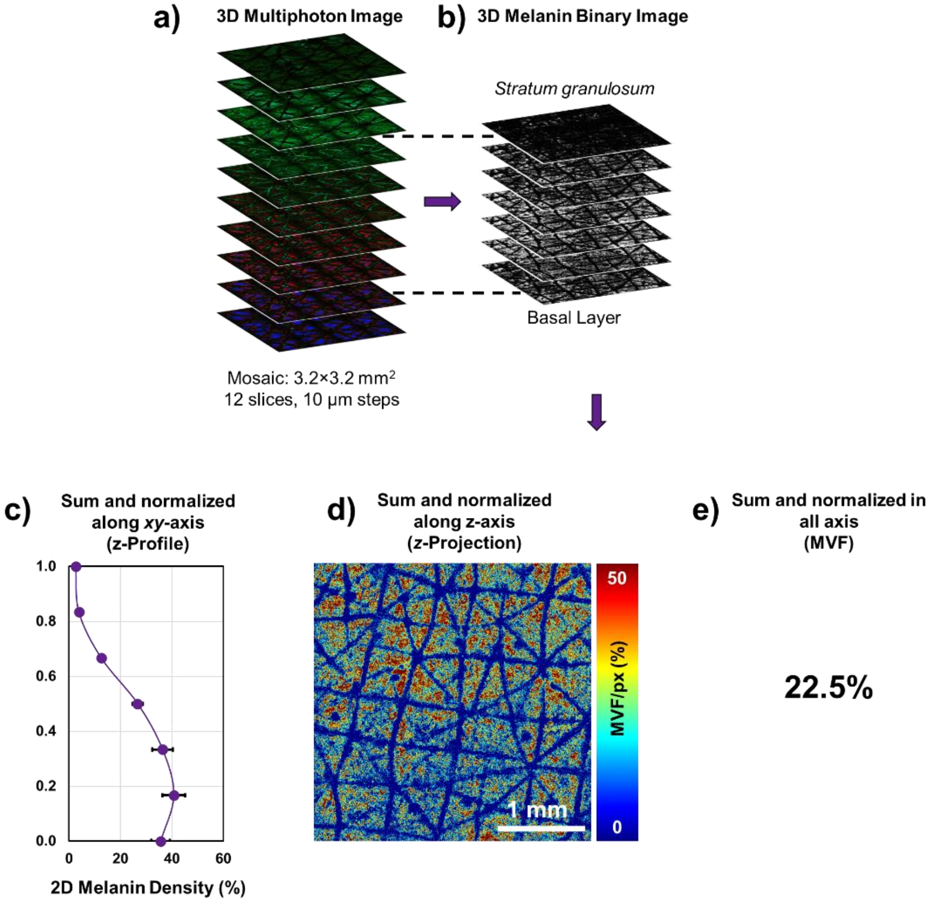

Figure S3. Schematic diagram for the data analysis. a) Raw 3-channel MPM volumetric image. b) The corresponding melanin binary volumetric image from the *stratum granulosum* to the basal layer. c) The resulting *z*-profile when the sum of (b) is taken along the *xy*-axis and normalized against the total number of pixels for each slice. d) The resulting *z*-projection when the sum of voxels in (b) is taken and normalized against the total number of pixels for each voxel. e) MVF is calculated by taking the global sum of (b) and divided by its total number of pixels.

Table S1a. Analysis of Variance (ANOVA) for the average global MVF of volar forearm

Source	SS	df	MS	F	Prob > F
Columns	2.215	4	0.554	147.140	1.044E-51
Error	0.583	155	0.004		
Total	2.798	159			

Table S1b. *P*-values from Tukey-Kramer post-hoc analysis for ANOVA from Table S1a

Type	II	III	IV	V
I	4.389E-02	1.881E-06	0.000E+00	0.000E+00
II		1.062E-01	1.239E-17	0.000E+00
III			4.158E-09	0.000E+00
IV				0.000E+00

Table S2a. Analysis of Variance (ANOVA) for the average global MVF of dorsal forearm

Source	SS	df	MS	F	Prob > F
Columns	3.670	4	0.917	235.709	8.684E-65
Error	0.603	155	0.004		
Total	4.273	159			

Table S2b. *P*- values from Tukey-Kramer post-hoc analysis for ANOVA from Table S2a

Type	II	III	IV	V
I	1.355E-08	8.104E-16	0.000E+00	0.000E+00
II		1.861E-01	0.000E+00	0.000E+00
III			1.738E-14	0.000E+00
IV				0.000E+00

Table S3. Summary of two-sample unpaired t-test for the comparison of average MVF from volar and dorsal forearm

Type	Mean MVF ± S.D. (%) Volar			Mean MVF ± S.D. (%) Dorsal			*P*-value
I	6.78	±	6.32	9.67	±	9.67	1.637E-01
II	11.04	±	3.41	19.12	±	3.41	1.752E-05
III	14.77	±	4.03	22.52	±	4.03	1.307E-11
IV	24.35	±	5.79	34.84	±	5.79	7.128E-12
V	39.83	±	6.31	53.59	±	6.31	2.175E-11

Table S4a. Analysis of Variance (ANOVA) for the measurements shown in Figure 5 in main text

Source	SS	df	MS	F	Prob > F
Columns	26.39	2	13.1928	0.8702	0.4317
Error	363.86	24	15.1608		
Total	390.24	26			

Table S4b. *P*- values from Tukey-Kramer post-hoc analysis for ANOVA from Table S1a

Trial	T2	T3
T1	0.475	0.996
T2		0.524

Table S5. Sample size estimate as a function of FOV size for the detection of 10 to 25% change in the MVF values based on two-sample unpaired t-test (Power: 80% and significance level: 5%)

FOV (mm^2)	Mean ± S.D. (%)			10%	15%	20%	25%
0.25^2	53.6	±	11.6	74	33	19	12
0.29^2	53.6	±	10.2	57	26	15	10
0.46^2	53.6	±	7.7	33	15	9	6
0.81^2	53.6	±	6.3	22	10	6	4
1.62^2	53.6	±	5.0	14	7	4	3
